# Life cycle design of polyhydroxyalkanoates (PHA)

**DOI:** 10.1093/nsr/nwaf517

**Published:** 2025-11-21

**Authors:** Simian Sun, Shimao Yang, Yu Qiu, Jun Ding, Wanze Wang, Fuqing Wu, Guo-Qiang Chen

**Affiliations:** School of Life Sciences, Tsinghua University, Beijing 100084, China; School of Life Sciences, Tsinghua University, Beijing 100084, China; School of Life Sciences, Tsinghua University, Beijing 100084, China; School of Life Sciences, Tsinghua University, Beijing 100084, China; School of Life Sciences, Tsinghua University, Beijing 100084, China; School of Life Sciences, Tsinghua University, Beijing 100084, China; Center for Synthetic and Systems Biology, Tsinghua University, Beijing 100084, China; School of Life Sciences, Tsinghua University, Beijing 100084, China; Center for Synthetic and Systems Biology, Tsinghua University, Beijing 100084, China; Tsinghua-Peking Center for Life Sciences, Beijing 100084, China; MOE Key Lab of Industrial Biocatalysis, Department of Chemical Engineering, Tsinghua University, Beijing 100084, China; State Key Laboratory of Green Biomanufacturing, Tsinghua University, Beijing 100084, China

**Keywords:** poly-β-hydroxybutyrate, polyhydroxyalkanoates, circular economy, life cycle assessments, next generation industrial biotechnology, NGIB, *Halomonas*

## Abstract

The global plastic crisis demands sustainable polymer design and production across the full life cycle. Polyhydroxyalkanoates (PHAs), a family of biodegradable polyesters produced by microorganisms, provide a representative model for circular material development and applications. This review summarizes advances in microbial chassis engineering, seawater-based *Halomonas* biomanufacturing, and low-energy downstream processing that together reduce freshwater use, energy input, and process complexity. The structural versatility of PHA supports applications ranging from compostable packaging to long-term biomedical devices. End-of-life options, including biodegradation, anaerobic digestion, and chemical recycling, enable efficient material recovery, and reintegration into natural carbon cycles. Life cycle assessments consistently show reductions in greenhouse-gas emissions, fossil-resource dependence, and marine eutrophication relative to conventional plastics. Remaining challenges include lowering production costs, improving material performance, and developing standardized biodegradation and circular-economy frameworks. Integration on synthetic biology, materials science, and industrial ecology help shape design principles for sustainable PHA-based polymer systems.

## Teaser

This review traces the progress in the life cycle design of polyhydroxyalkanoates (PHA), bioplastics mass-produced by engineered bacteria using sustainable resources, for a circular bioeconomy.

## INTRODUCTION

With the continuous development of modern society and the global economy, the demand for single-use plastics has surged, significantly accelerating the pace of plastic production. Recent studies estimate that global plastic consumption will increase several-fold in the coming decades, largely driven by the use of virgin fossil-based raw materials. This dramatic expansion, combined with the persistence and non-biodegradability of conventional plastics, poses escalating threats to ecosystems and human health due to long-term plastics environmental accumulation and microplastic pollution. This surge in plastic production, coupled with the inherent non-biodegradability of conventional plastics, exacerbates environmental and public health risks [1,2]. Addressing this issue requires comprehensive strategies, including reducing plastic production, improving waste management, and advancing research into biodegradable alternatives. To reconcile the dual challenges of escalating plastic demand and environmental sustainability, transitioning from linear (produce-use-dispose) to circular (design-regenerate-redesign-reuse) production models has become imperative. Biodegradable polymers like polyhydroxyalkanoate (PHA) exemplify this paradigm shift. Biodegradable plastics are primarily composed of bioplastics derived from renewable resources. These bioplastics are synthesized from monomers extracted or synthesized from biomass compounds, such as plant-based sources, and are polymerized to replace petroleum-based plastics. PHA produced via microbial fermentation serves as the best example of a circular material [3].

PHAs are a class of polyesters synthesized by microorganisms as intracellular carbon and energy storage compounds as well as stress protectants under conditions of excess carbon availability, nutrient imbalance and other growth stresses, where they are accumulated as discrete inclusion bodies [4]. Recognized as a biodegradable alternative to conventional plastics due to their comparable material properties, PHAs have garnered extensive research interest owing to their exceptional attributes, including superior biocompatibility, environmentally benign degradation products of non-toxicity, and chemically tunable physicochemical characteristics [2]. Crucially, the microbial biosynthesis of PHAs align with a life cycle design (design-regenerate-redesign), contrasting with the conventional plastic production model (produce-use-dispose) [5]. This biological production strategy leverages microbial metabolic engineering to achieve carbon-neutral synthesis, positioning PHA as a cornerstone material for circular bioeconomy initiatives while addressing critical challenges in plastic pollution and resource sustainability.

**Figure 1.**
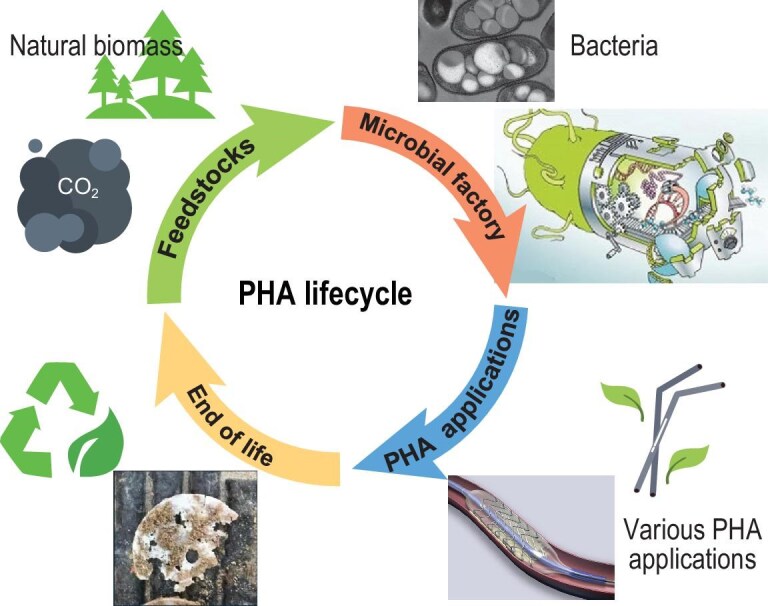
Life cycle of sustainable PHA polymers [2–8]. PHA polymers originated from renewable or waste-derived feedstocks, are biosynthesized by microbial cells, used across various fields, recovered through responsible end-of-life strategies, and ultimately regenerated into new materials.

Life cycle assessment (LCA) is a methodological framework specifically designed to evaluate the environmental impacts associated with all stages of a product’s life cycle (cradle to grave), encompassing raw material extraction (cradle), production, utilization, and end-of-life management including disposal, recycling, or biodegradation (grave) [6]. The life cycle design of PHA polymers constitutes a systematic design philosophy that holistically integrates sustainability considerations throughout the PHA production chain—from biomass feedstock acquisition and bioprocess implementation, through product application, to ultimate waste management and environmental degradation (Fig. 1). Following the conceptualization of green chemistry principles in the early 1990s, researchers subsequently developed comprehensive LCA protocols, both frameworks emphasizing environmental stewardship and sustainable development [6]. PHA polymers can demonstrate favorable performance in green design metrics and LCAs, particularly regarding fossil fuel displacement and reduced global warming potential, provided that each step of the production chain is carefully optimized [7].

The PHA polymers are synthesized from renewable, recyclable natural biomass, or other low-carbon feedstocks and are managed in an environmentally responsible manner at end-of-life through recycling or biodegradation. These microbially synthesized biopolymers exhibit inherent sustainability across their entire life cycle, aligning with the circular economy paradigm by enabling closed-loop material flows [8]. The life cycle of sustainable PHA polymers spans from feedstock acquisition to closed-loop regeneration (Fig. 1).

In this review, we employ a cradle-to-grave life cycle design framework to holistically evaluate PHA as a sustainable biopolymer. Beginning with microbial chassis engineering for biosynthesis, we analyze strategies to optimize carbon source selection (e.g. waste-derived feedstocks) and fermentation processes that reduce freshwater consumption by up to 70% [9]. Subsequent sections integrate downstream extraction innovations, such as self-flocculating microbial cells, which lower energy use by 40% and solvent inputs by 35%, directly aligning with circular production goals [10]. The work further establishes structure-function relationships between PHA monomer compositions (e.g. short-chain vs medium-chain) and their life cycle performance—linking rapid biodegradability (<60 days in soil) to single-use packaging design, while tailoring slow-degrading variants for durable medical implants [11]. Post-consumer environmental impacts are quantified through standardized LCA metrics, revealing a 58% reduction in global warming potential (2.1 kg CO_2_-eq/kg PHA) compared to conventional plastics [12]. Finally, we propose closed-loop regeneration systems, where degraded PHA byproducts re-enter production cycles as carbon sources, achieving near-zero waste thresholds in pilot studies [13].

## SUSTAINABLE MICROBIAL PLATFORMS FOR PHA PRODUCTION

Building on advances in microbial engineering and bioprocess optimization, robust microbial platforms now provide the foundation for designing efficient and environmentally favorable PHA production processes [14]. These platforms enable sustainable production of diverse chemical compounds, natural products, and microbial polyesters such as PHA [14].

The structure and composition of PHA synthesized by microorganisms depend on the carbon sources and metabolic pathways employed. These variations influence polymer properties, process efficiency, and environmental impacts, that are critical considerations in life cycle assessment [14–17].

Numerous microorganisms, including bacteria, archaea, and fungi, are capable of producing PHA [15]; however, bacterial strains remain the primary hosts for commercial production. *Cupriavidus necator* (*C. necator*) is a well-established Gram-negative strain widely used for industrial production of poly-β-hydroxybutyrate (PHB), poly((R)-3-hydroxybutyrate-co-4-hydroxybutyrate) (P3HB4HB), and poly((R)-3-hydroxybutyrate-co-(R)-3-hydroxyvalerate) (PHBV) [16–18]. This strain can achieve cell dry weights (CDWs) up to 200 g/L with PHB accumulation reaching 80% of CDW within 72 hours [19], and possesses RuBisCO genes enabling autotrophic growth via CO_2_ fixation [20].

At Tsinghua University, we have established an industrial PHA production platform using *Halomonas bluephagenesis* as a chassis organism, exemplifying Next-Generation Industrial Biotechnology (NGIB) for cost-effective and scalable PHA manufacturing [10]. *Halomonas bluephagenesis* TD01 is a halophilic strain suitable for open (unsterile) fermentation, achieving 80 g/L CDW with 80% PHB content in <56 hours under glucose-fed batch cultivation in a saline medium [21]. Recent advances in morphological engineering and targeted genetic modifications have further improved *Halomonas* spp., enabling CDW of 149 g/L with 82% PHB during a 44-hour fermentation in a 5000-L bioreactor [22].

Collectively, these developments demonstrate that tailored microbial platforms can significantly enhance the efficiency, scalability, and environmental performance of PHA production, providing a seamless transition to life cycle assessment of PHA materials [14–22].

## NEXT-GENERATION INDUSTRIAL BIOTECHNOLOGY (NGIB)

**Figure 2.**
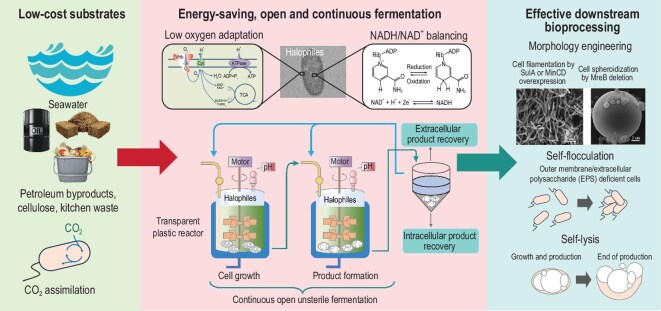
Next-Generation Industrial Biotechnology (NGIB) for sustainable polyhydroxyalkanoate (PHA) production by *Halomonas* [1,17,24,26]. Strategies applied using *Halomonas*-based NGIB for eco-efficient PHA biomanufacturing. Key aspects include: (1) Broad substrate utilization—metabolic engineering enables the efficient bioconversion of diverse, low-cost carbon sources (e.g. seawater, petroleum byproducts, lignocellulosic biomass, and food waste) into PHA. (2) Continuous fermentation—the halotolerance and innate resistance to contamination of *Halomonas* support uninterrupted, high-cell-density cultivation in open, unsterile reactors. Process efficiency is further enhanced by heterologous expression of *Vitreoscilla* hemoglobin to improve oxygen uptake and by modulation of the intracellular NADH/NAD^+^ balance. (3) Efficient downstream processing—morphological engineering strategies are applied to modulate cell volume and intracellular PHA granule size; cell surface engineering (e.g. deletion of outer membrane components and EPS biosynthesis genes) facilitates spontaneous flocculation; and phage-derived lysis systems enable streamlined cell disruption and product release.

Traditional industrial biotechnology relies on conventional microorganisms such as *E. coli, Bacillus* spp., *Corynebacterium glutamicum*, yeasts, etc. for chemicals, fuels, or PHA production. However, this approach requires energy-intensive sterilization processes, and complex downstream operations, leading to significant freshwater consumption and high production costs, which have reduced the economy of the large-scale application of PHA [17,21]. In recent years, NGIB based on halophilic microorganisms has emerged as a transformative solution for the sustainable life cycle design of PHA: by integrating halotolerant chassis cells, open continuous fermentation, and intelligent downstream processing technologies, NGIB offers a revolutionary approach to PHA production (Fig. 2) [23,24]. Halophilic microorganisms, especially *Halomonas*, leverage their unique characteristics to efficiently synthesize PHA in non-sterile seawater media. This approach enables wastewater recycling and waste salt reuse, significantly reducing resource consumption and carbon emissions (Table 1) [25].

**Table 1.** Comparison of NGIB and current industrial biotechnology (CIB) [10,14–22].

Categories	NGIB	CIB
Freshwater consumption	Low freshwater consumption	High freshwater demand
Food based substrates	Cellulose, kitchen waste, CO_2_ or derivatives	Glucose or corn
Energy intensity	Low	High
Sterile operation	No	Yes
Continuity of process	Continuous	Batch or Fed-batch
Complexity of process	Low	High
Wastewater treatment	Simple recycle	Complex
Equipment	Steel, glass or plastic bioreactors	Stainless steel
Production cost	Low	High

As for upstream microbial cell optimization, metabolic engineering expands the substrate utilization range of *Halomonas* to include low-cost carbon sources such as lignocellulosic hydrolysates, food waste, and CO_2_ derived from agricultural and industrial wastes [27,28]. In midstream cultivation, open continuous fermentation and high-density cultivation technologies drastically reduce energy consumption during production. Last, in downstream processing, morphological engineering enhances the auto-settling properties of microbial cells. Combined with intracellular PHA granule size regulation, self-flocculation, and self-lysis systems, these innovations simplify separation processes, enabling efficient PHA extraction without centrifugation (Fig. 2) [29,30].

### Substrate utilization

*Halomonas* have emerged as an excellent chassis for NGIB due to their exceptional salt tolerance, resistance to contamination, convenient scale-up, and broad substrate utilization (Fig. 2) [31,32]. Through systematic metabolic engineering and fermentation process optimization, *Halomonas* demonstrates remarkable advantages in raw material utilization, effectively reducing resource consumption, minimizing environmental pollution, and enhancing both economic and social benefits (Fig. 2) [33].

### Utilization of diverse low-cost carbon sources

*Halomonas* can metabolize a broad spectrum of low-cost carbon sources, including industrial and agricultural waste, thereby significantly reducing the overall cost of biomanufacturing. For instance, *Halomonas campaniensis* LS21 secretes extracellular enzymes including amylases, lipases, and cellulases, which enable continuous and stable fermentation for >65 days in a non-sterile seawater medium supplemented with mixed substrates such as cellulose and kitchen waste [34]. Similarly, engineered *H. bluephagenesis* TD01, harboring the essential gene *ompW* followed by the PHA synthesis *phaCAB* operon, exhibits enhanced adaptability to food waste, overcoming batch-to-batch compositional variability and enabling stable PHA production [34]. Furthermore, petroleum and coal-derived chemical byproducts (e.g. CO_2_, formate, methanol, acetate), plastic degradation intermediates (e.g. 1,4-butanediol), lignocellulosic hydrolysates, and agricultural residues (e.g. defatted rice bran, rice milling wastewater) have been successfully repurposed as alternative carbon sources for *Halomonas* [35–39], thereby expanding feedstock diversity (Fig. 2).

In lignocellulose utilization, metabolic engineering of *H. bluephagenesis* has enabled the efficient conversion of lignocellulosic hydrolysate into PHB. By introducing xylose-specific transporters and three distinct xylose utilization pathways—xylose isomerase (XI), xylulose-1-phosphate (X-1-P), and ribulose-1-phosphate (R-1-P)—the strain achieved a CDW of 62 g/L and PHB content of 67% in a 7 L fermenter using lignocellulosic hydrolysate [40,41]. Additionally, *Halomonas cupida* J9, employing a glucose/xylose co-feeding strategy, attained 7.0 g/L CDW and 2.45 g/L PHA titer in a 5-L bioreactor using corn stover hydrolysate under open, non-sterile fermentation conditions [32,42].

### Metabolic engineering for enhanced substrate utilization efficiency

To overcome the inherently limited ability of *Halomonas* strains to degrade complex substrates, metabolic engineering strategies have been employed to enhance their degradation and biosynthetic capacities. Engineered *H. bluephagenesis*, for example, secretes amylases and glucosidases enabling the direct utilization of corn starch for PHB production, resulting in 10 g/L CDW and 51% PHB content in shake-flask cultures [32,42]. In acetate utilization, adaptive laboratory evolution (ALE) of *H. bluephagenesis* B71 improved both acetate tolerance and PHB biosynthesis efficiency, yielding 70 g/L CDW and 50 g/L PHB in fed-batch fermentation [43].

Recent engineering of CO_2_ fixation further broadens the substrate spectrum of *Halomonas*. Overexpression of *Escherichia coli* phosphoenolpyruvate carboxykinase (*PckA*) in *H. bluephagenesis* enhanced CO_2_ assimilation, increasing PHB content from 73% to 78%, and PHB titer from 7.2 g/L to 8.7 g/L [44]. Additionally, certain species such as *Halomonas rowanensis* employ the reductive tricarboxylic acid (rTCA) cycle for CO_2_ fixation, providing novel insights into carbon-neutral biotechnologies [44].

### Industrial potential and economic viability

The inherent halotolerance and high pH tolerance of *Halomonas* enable non-sterile continuous fermentation, thereby drastically reducing energy consumption and equipment costs (Table 1). Open, non-sterile fermentation employing *Halomonas* spp. eliminates energy-intensive sterilization steps, resulting in a 60% reduction in freshwater consumption and a 45% decrease in operational costs compared to traditional batch processes [10]. This approach aligns with industrial scalability while simultaneously minimizing carbon footprints (2.1 kg CO_2_-eq/kg PHA vs 5.8 kg CO_2_-eq/kg poly(ethylene terephthalate) (PET)) [45]. Phenotypic microarray analysis revealed that *H. bluephagenesis* TD01 is capable of utilizing 140 out of 190 tested compounds, including sodium acetate, glycerol, ethanol, and lactate, highlighting its metabolic versatility [31]. Notably, *H. bluephagenesis* TD01 exhibits robust tolerance to high concentrations of salts, with growth rates decreasing by only 56%, 53%, and 47% under 75 g/L sodium acetate, 100 g/L succinate, and itaconate, respectively (Table 1) [31]. When using acetate as a substrate, engineered *Halomonas* strains not only synthesize PHB efficiently but also produce 121 g/L mevalonic acid (MVA) via an introduced MVA pathway under open culture conditions [46]. These technologies reduce feedstock costs while valorizing waste streams, aligning with circular economy principles by mitigating methane emissions [47].

Despite notable progress, further research is needed to elucidate the carbon fixation pathways and complex substrate metabolism in *Halomonas*. For instance, the thiosulfate-driven chemolithoautotrophic pathway in *Halomonas* remains poorly characterized, identifying and characterizing the associated genes could unlock applications for inorganic substrate utilization [48]. Additionally, the development of multi-substrate co-utilization strategies, optimizing metabolic flux distribution, and diversifying products (e.g. medium- and short-chain PHA copolymers, small-molecule chemicals) remain critical priorities [49].

Through rational design and engineering, *Halomonas* species have achieved highly efficient conversion of wastes into value-added products, reducing resource consumption, environmental burden, and production cost [50]. Advances in *Halomonas* engineering and bioprocess design are enabling closed-loop polymer systems, where PHA is synthesized from waste-derived carbon sources and ultimately returns to the environment through biodegradation [51].

### PHA production process and conditions

PHAs, as environmentally friendly and biodegradable materials, have garnered significant attention due to their potential in sustainable design throughout the entire product life cycle [52]. However, conventional biomanufacturing processes for PHA production are hindered by challenges such as excessive freshwater consumption, energy-intensive sterilization procedures, and complex process equipment, resulting in resource inefficiency and high production costs [53]. Recent advances in engineering halophilic bacteria as chassis cells have offered transformative solutions for PHA production, leveraging their unique adaptability to extreme environments and their amenability to synthetic biology modifications [54]. Here we highlight the application innovations of halophilic bacteria across key dimensions, including water conservation, energy efficiency, continuous fermentation, and high-cell-density cultivation, highlighting their impact on advancing the sustainable manufacturing of PHA [55].

### Synergistic optimization of resource utilization and energy consumption

Traditional PHA production depends substantially on freshwater, primarily for growth media, equipment cleaning, temperature control, washing, and cooling processes. Halophilic bacteria, such as *Halomonas* species, provide a distinct advantage of using seawater (or artificial seawater) to replace fresh water, thereby reducing reliance on freshwater resources (Fig. 2) [34]. Additionally, their metabolic pathways allow for the simultaneous synthesis of PHA and high-value-added products like ectoine, 3-hydroxypropionate, enzymes, proteins, and chemicals, further enhancing resource utilization efficiency.

In terms of energy consumption, the halotolerance of halophilic bacteria (pH >8.5) enables them to thrive in open environments, naturally inhibiting contamination by other microorganisms [13]. This characteristic eliminates the need for high-temperature sterilization steps in traditional fermentation processes, simplifying workflows and lowering equipment demands for thermal resistance, thereby significantly lowering energy consumption [21]. Furthermore, metabolic reprogramming of halophilic bacteria through synthetic biology approaches has enhanced their adaptability to low-oxygen conditions [56]. For instance, Chen *et al.* demonstrated a two-fold increase in CDW under low-oxygen conditions by introducing the *vgb* gene (encoding hemoglobin from *Vitreoscilla*) and utilizing the twin-arginine translocation (Tat) pathway to localize it to the periplasm [57,58]. Combined with cofactor ratio regulation (e.g. NADH/NAD^+^ balance) and directed evolution, the PHA synthesis efficiency of halophilic bacteria under low-oxygen conditions has been systematically improved, offering a practical approach for reducing energy consumption associated with air compressors [57,58].

### Continuous fermentation for PHA production

Traditional batch fermentation processes are frequently interrupted for culture processing and sterilization of reactors, leading to lengthening period and labor costs. The remarkable environmental adaptability of halophilic bacteria provides a foundation for the application of continuous fermentation technology. This technology maintains a steady state within the reactor by continuously supplying fresh medium and discharging fermentation broth, keeping microorganisms in the logarithmic growth phase, thereby improving productivity. Studies have shown that continuous fermentation reduces freshwater demand and wastewater treatment loads through the recycling of culture media [34]. *Halomonas* has been successfully tested in non-sterile conditions at laboratory scale (7 L) and large scale (1–5 m^3^), demonstrating its industrial feasibility with continuous cultivation [21].

In traditional processes, high concentrations of products may inhibit microbial metabolism, whereas in continuous mode, products are dynamically diluted with the flow of fermentation broth, maintaining stable production rates [59]. Moreover, the high-salinity and alkaline environment created by halophilic bacteria naturally prevents contamination by other microorganisms, providing a unique safeguard for open operations and further reducing equipment and operational complexity [60].

### Scaling-up of high-density cultivation

High-density cultivation is a key strategy for improving PHA production efficiency by increasing cell density per unit volume, which improves productivity and lowers downstream separation costs. A derivative of *Halomonas* TD01 achieved a CDW of 100 g/L with a PHA content of 60.4% during open continuous fermentation [61,62]. This breakthrough was achieved through iterative ‘mutation-screening’ strategies to optimize metabolic pathways, such as reprogramming redox networks to balance intracellular metabolic fluxes, thereby significantly improving substrate conversion efficiency [58].

Scale-up production validation further underscores the industrial potential of this technology. *Halomonas* has been successfully scaled up to 225 m^3^ fermenters for PHA production, marking a successful transition from laboratory to industrial-scale applications [61]. Future efforts should focus on developing more stable mutants to enhance the robustness of strains under extreme conditions such as high temperature, low oxygen, and low salinity, thereby addressing the reduced complexities of large-scale production.

The application of *Halomonas* as chassis cells has systematically restructured PHA production processes by integrating seawater utilization, sterilization-free protocols, continuous fermentation, and high-density cultivation. These innovations have significantly reduced resource consumption and carbon emissions, thereby providing technical support for cost optimization and aligning with the goals of sustainable life cycle design. While the industrial production system based on *Halomonas* has established a foundation for large-scale application, further research is required in areas such as microbial stability, precise metabolic pathway regulation, and intelligent fermentation processes for stabilizing PHA structures and molecular weights. With the deep integration of synthetic biology and process engineering, *Halomonas*-based PHA production is poised to become a paradigm in the biomanufacturing sector, driving a comprehensive upgrade in the biodegradable materials industry.

### Downstream processing

Bacterial morphological engineering holds significant potential in enhancing microbial downstream processing efficiency and intracellular product accumulations. Studies [22,62,63] revealed that targeted genetic modifications of cell shape-determining genes (e.g. *ftsZ, sulA*, and *mreB*) enables microbial self-sedimentation characteristics. In *Halomonas* spp., *mreB* knockout converts rod-shaped cells into spherical forms, while *ftsZ* inactivation induces filamentous cell shape formation. Both modified morphologies substantially improve natural sedimentation rates by >3-fold compared to wild-type strains through increased cell volume or modified hydrodynamic properties [22,64]. Notably, this morphology-driven self-separation strategy has been successfully integrated with non-sterile continuous fermentation for PHA production by *H. bluephagenesis*, achieving a 69%–82% increase in extraction efficiency and reduction in purification costs compared to conventional centrifugation-based methods [62,64]. The enhanced processability arises from distinct mechanisms: elongated cells exploit increased surface-area-to-volume ratios to enhance gravitational sedimentation, whereas spherical morphologies maximize cell volumes with enhanced weights for accelerating precipitation. This principle extends beyond cellular morphology to intracellular product architecture [65]. In *H. bluephagenesis*, the separation challenge posed by small PHA granules can be overcome by engineering both granule size and cell morphology. The phasin proteins PhaP1, PhaP2, and PhaP3 play distinct roles in controlling PHA granule morphology: *phaP1*-knockout strains produced a single large PHA granule filling almost the entire cellular space, whereas *phaP2*/*phaP3* deletions resulted in moderately enlarged granules, indicating their differential involvement in granule stabilization and size regulation [30]. Concurrent overexpression of cell division inhibitors MinC and MinD created enlarged cellular compartments accommodating PHA granules up to 10 μm in diameter [30]. These findings demonstrate that PHA granule size is fundamentally constrained by host cell dimensions. By synergistically combining morphological expansion with granule size regulation strategies, the study achieved ultra-large PHA granules optimized for mechanical separation [62,64]. Future directions include multiplex gene regulation to synchronize morphology optimization with product synthesis and scale-up studies for process scale up [66]. This paradigm shift toward a ‘design-for-separation’ microbial chassis could revolutionize sustainable bioproduction by simultaneously enhancing both upstream productivity and downstream recoverability [67].

Engineered self-flocculating strains reduce dependence on intensive centrifugation, reducing downstream energy consumption by ∼40% and solvent inputs by 35%. Such innovations lower purification costs while enhancing process sustainability [64]. Parallel progress has been made in programmed cell lysis systems: chromosomal integration of phage SRRz lytic genes with synthetic ribosome binding sites created stress-induced ‘self-disruption’ strains. These engineered systems enable controllable product release through either solvent induction during processing or spontaneous lysis upon nutrient depletion, effectively obviating the need for mechanical disruption [68]. Future implementation of these approaches in continuous fermentation platforms promises to establish fully automated, centrifugation-free bioprocessing pipelines for industrial biopolymer production.

The highly effective strategy of combining synergistic integration of morphological engineering, extracellular polysaccharide (EPS) modification, and phage-based lysis systems represents an effective approach to downstream bioprocessing [22,68,69]. By coupling cell shape optimization with EPS-deficient self-flocculation, these strategies reduced dependence on intensive centrifugation while enhancing product extraction efficiency via enhanced cell permeability. Cell autolysis systems further streamline intracellular product release, collectively reducing purification costs [68]. Future efforts should prioritize multiplexed control of these parameters in continuous fermentation platforms to establish fully automated, centrifugation-free workflows. This ‘design-for-separation’ paradigm, synchronizing upstream production with downstream recoverability, promises to advance industrial-scale biomanufacturing of sustainable PHA production via energetically and economically optimized processes [67].

## CHEMICALLY SYNTHESIZED PHA

PHA can also be synthesized through chemocatalytic routes. The dominant strategy is the ring-opening polymerization (ROP) of lactones and related cyclic esters [70]. ROP of four-membered β-butyrolactone (β-BL) and eight-membered diolides (8DL) has enabled the stereospecific synthesis of isotactic P3HB with controllable molecular weight and dispersity [71]. Catalyst and monomer design therefore play central roles in determining the stereoregularity of chemically synthesized PHA. Recent advances also include a biomimetic synthesis of P4HB using C4 diol and air via cooperative catalysis in the ROP of γ-butyrolactone, providing an efficient and mild route to P4HB analogues [72]. Furthermore, stereo-meric polymerization strategies have enabled the conversion of natural (R)-P3HB into all enantiomerically pure di-isotactic PHA diastereomers, overcoming the long-standing limitation of biological pathways that afford a single stereo-configuration [73].

A major advantage of chemical synthesis is its ability to access polymer architectures that are difficult to generate via cellular metabolism, including PHA-based block copolymers and topologically complex structures. A wide range of methodologies has been reported for the synthesis of PHA block copolymers from functionalized PHA segments [74]. In addition, α,α-disubstituted PHA platforms have recently been developed to simultaneously address mechanical brittleness, thermal instability, and the need for closed-loop chemical recyclability, yielding materials that are tough, melt-processable, and chemically circular [75].

Several recent authoritative reviews have provided comprehensive summaries of these developments, including advances in monomer design, catalytic control, chemical recyclability, and functional PHA architectures [76–78]. Together, these advances establish chemically synthesized PHA as a versatile complement to biological PHA, particularly in structural precision, functional tunability, and circularity.

### Critical comparison between synthetic PHA and biological PHA

Chemical and biological routes to PHA represent complementary strategies that differ in feedstock origin, process characteristics, and accessible polymer structures. Biological PHA are synthesized intracellularly from renewable or waste-derived carbon sources, embedding them within a circular bioeconomy. Although synthetic PHAs have traditionally relied on petrochemical monomers, modern developments demonstrate that synthetic PHA can also be bio-derived, for example through the use of bio-based succinate and other renewable platform chemicals as precursors for polyester monomers [79,80]. Recent comprehensive analyses further emphasize the convergence between biological and chemical routes enabled by renewable monomer platforms [76,77].

Biological PHA production occurs in aqueous media under mild temperatures and pressures. Synthetic PHAs, in contrast, are produced via catalytic polymerization. While early synthetic routes sometimes required stringent conditions, many modern chemocatalytic processes now operate under ambient or near-ambient temperatures and even in solvent-free bulk systems, utilizing low loadings of tunable molecular catalysts and requiring minimal post-polymerization purification [77,78]. Therefore, it is not accurate to generalize chemical synthesis as inherently complex, energy-intensive, or hazardous.

The two approaches offer distinct advantages. Biological pathways efficiently yield high-molecular-weight polymers, but often with broad molecular-weight distributions and limited control over block structure or stereochemistry. In contrast, synthetic PHA can achieve precise control over molecular weight, dispersity, tacticity, and block sequence, enabling fine-tuning of mechanical and thermal properties [74]. Advances in stereocontrolled ROP and functional monomer design have further expanded the diversity of accessible PHA architectures [76,78]. Biological PHAs benefit from mature fermentation platforms and the ability to incorporate waste-derived feedstocks, while synthetic PHAs remain more dependent on catalyst cost and monomer availability at scale.

Looking forward, an important trend is the integration of biological and chemical strategies. Biologically produced chiral monomers or oligomers from renewable feedstocks can be used as building blocks for chemocatalytic polymerization, allowing the creation of PHA materials with sophisticated architectures and enhanced performance while maintaining environmental sustainability [77,79,80]. Such hybrid approaches combine the complementary strengths of both routes and represent a promising direction for next-generation PHA manufacturing.

## STRUCTURES, CLASSIFICATION, AND APPLICATIONS OF PHA

### Diverse structures of PHA

The constituent monomers of bio-PHA are chiral R-hydroxy fatty acids, and the carboxyl group of the monomer undergoes a condensation reaction with the hydroxyl group of the adjacent monomer to form ester bonds which are further polymerized into PHA [81]. Variations in monomer carbon chain length, polymerization pattern, alkyl side chain (R) structure, and molecular weight collectively underpin the structural diversity of PHA biopolymers [2].

Depending on the length of the monomer carbon chain, PHA can be categorized into three main groups: short-chain length PHA (SCL-PHA), medium-chain length PHA (MCL-PHA) and long-chain length PHA (LCL-PHA) [82,83]. Typically, SCL-PHA contains monomers with 3–5 carbon atoms, while MCL-PHA with carbon chain lengths ranging from 6 to 14 carbon atoms. Monomers of LCL-PHA with more than 14 carbon atoms in length are rarely produced by microorganisms [84]. According to the difference of monomer types and arrangements, PHA can also be divided into homo- and co-polymers, the latter can be further subdivided into random copolymers and block copolymers. In addition, functional polymers, containing double or triple bonds, carbonyl, epoxy, cyano, phenyl, and/or halogen groups on the polymer side chains, could be modified chemically via these functional groups to provide novel functionalities [67]. Meanwhile, PHA can also be modified biologically by introducing new functional monomers and modified physically by blending with natural raw materials or other synthetic biodegradable polymers to produce novel polymers with expected variations in their molecular weights and functions, altering their characteristics such as thermal mechanical properties, hydrophilicity, surface structure, and rate of degradation [85].

Since the discovery of PHA in microbial cells nearly a century ago, more than 150 different monomers have been identified [82]. However, only six representative PHAs have been successfully produced at an industrial scale [86], including PHB; its copolymers of 3-hydroxyvalerate (3HV) termed PHBV; 3HB and 3-hydroxyhexanoate (PHBHHx); 3HB and 4-hydroxybutyrate (P34HB); poly(4-hydroxybutyrate) (P4HB); and terpolymers of 3-hydroxybutyrate, 3-hydroxyvalerate, and 3-hydroxyhexanoate (PHBVHHx). With ongoing advances in microbial engineering and polymer chemistry, novel monomers and polymer forms continue to emerge, expanding the functional diversity and application spectra of PHAs as sustainable alternatives to conventional plastics [82]. For example, PhaBuilder, a start-up biotech company based in Beijing, China, has been able to produce >70+ PHAs with various monomer structures, arrangements, and molecular weights for commercial application developments (biophamily.com.cn).

### Properties of PHA

In general, PHB exhibits material properties of high hardness, poor flexibility and fast crystallization rate, whereas MCL-PHA displays lower stiffness, moderate elasticity, good ductility, slow crystallization rates and ease of thermal softening [85]. The thermal and mechanical properties of PHA—such as crystallinity, melting temperature, Young’s modulus, tensile strength, elongation at break, and glass transition temperature, can be finely tuned by changing the structures and ratios of short-, medium-, and long-length monomers. This enables the production of materials ranging from rigid, brittle to soft, ductile PHA for diverse applications [87].

PHAs synthesized by microorganisms possess inherent biodegradability, offering a viable alternative to synthetic plastics and contributing to the development of a circular economy. Based on the ester bonds in the main chain, PHAs can be decomposed by PHA depolymerases, lipases and esterases into their low molecular weight oligomers and monomers, and finally form CO_2_ and water by microorganisms [87]. The biodegradation rate of PHA is mainly determined by the affinity of degrading enzymes to the ester bonds in the chain segment, and the influencing factors include the type and composition of monomers, molecular weights, stereoregularity, crystallinity, surface morphology, and external environmental conditions such as temperature, moisture, and pH [88]. To date, >600 PHA depolymerases have been identified from various microorganisms capable of degrading PHA [89].

Some PHAs show excellent biocompatibility and biosafety, including blood compatibility, non-teratogenicity, non-carcinogenicity and non-toxicity for cells, tissues and organisms [90]. As one of the main degradation monomers of PHA, moderately acidic 3HB is an important component of natural ketone body produced by fatty acid oxidation, and is a significant alternative energy source for animals, including human [91]. Meanwhile, as an intracellular signal regulatory molecule, the potential therapeutic effects of 3HB on a variety of diseases have been reported, such as diabetes mellitus, atherosclerosis, colonic inflammation, and carcinogenesis [92–94]. PHA is also a family of biopolymers with optical activity and weak piezoelectric properties [85].

### Applications of PHA

**Figure 3.**
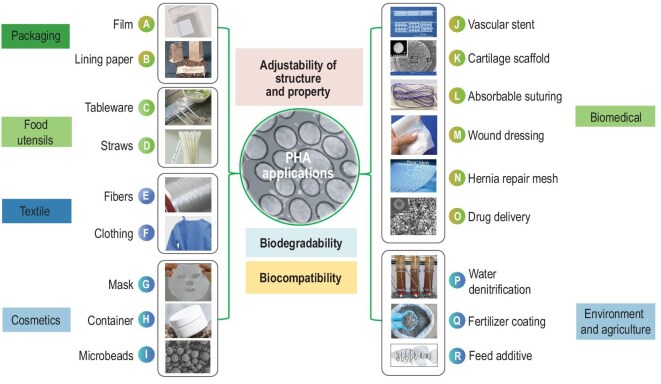
Overview of current PHA-based applications [65–68]. (A) Packaging film, (B) lining paper, (C) tableware, (D) straws, (E) fibers, (F) clothing, (G) container, (H) mask, (I) microbeads, (J) vascular stent, (K) cartilage scaffold, (L) absorbable suturing, (M) wound dressing, (N) hernia repair mesh, (O) drug delivery, (P) water denitrification, (Q) fertilizer coating (PHABuilder, https://eng.phabuilder.com/). (R) Feed additive. (B), (C), (D), and (G) are commercialized products from PHABuilder, (A), (E), (F), and (H) are products from Bluepha (https://www.bluepha.com/).

The structural versatility of PHAs leads to diverse material properties, which underpins their broad applicability across multiple industrial sectors [95]. PHA can find applications in industry including packaging, agriculture, medical, and cosmetic areas (Fig. 3, Table 2). Biodegradability is the pivotal characteristics supporting the realization of the PHA life cycle [19,52,95–105].

**Table 2.** Applications of PHA in various scenarios.

Fields	Types of PHA	Properties	Applications	References
Packaging	PHB, PHBV, PHBHHx	Flexibility, barrier properties of water and oxygen, compostable degradation, thermal stability	Shopping bags, lining for paper cups, blow molded jars and bottles	[18,95]
Disposable food utensils	PHB, PHBV	Mechanical strength, stiffness, flexibility, food-grade safety	Straws, cups, forks, spoon, food containers	[95]
Textile	PHBV, PLA/PHBV and PLA/PHB blends	Heat resistance and flexibility, tensile and thermal shrinkage properties	Surgical garments, upholstery, carpets	[18]
			Clothing, spinning fiber	[95,106]
Cosmetics	PHB, PHBV	Biocompatibility, adhesive properties	Surfactant masks for beauty, containers, cosmetic toner and facial pads	[95]
3D printing	P34HB, PHBV, PHBHHx	Thermal and mechanical strength, printability	Cultural and creative products, filaments, tissue engineering scaffolds	[99]
Wastewater treatment	PHB	Chemical resistance, durability, waste uptake	Nitrogen and organic waste removal	[98]
Agriculture	PHB, PHBV	Barrier properties, nutrient release control	Mulching films	[95]
			Coating for fertilizers, herbicides or insecticides for controlled-release, bacterial inoculants	[98]
Tissue engineering	PHB, P4HB, P34HB, PHBV, PHBHHx, PHBVHHx	Adjustable mechanical strength, porosity, tissue integration and biocompatibility	Absorbable suturing, plug and patch, hernia repair mesh	[100]
			Vascular stent	[101]
			Heart valve scaffold	[102,103]
			Electrospinning film	[104]
			Porous microspheres	[105]
			Scaffolds for tissue restoration and cell culture	[106]
Drug delivery	PHB, P34HB, PHBV, PHBHHx	Mechanical strength, biocompatibility, slower biodegradation	Drug release matrix	[86]

#### PHA as sustainable packaging materials

The designable mechanical properties with good O_2_, CO_2_ and water barrier properties, compostability, and lower carbon footprint of PHAs indicate that they can be suitable replacements for bulk packaging materials such as polyethylene (PE), polypropylene (PP), and PET, especially in the context of a circular economy (Fig. 3) [3,82]. PHAs are appropriate for short-term desired biodegradation applications that will encounter a compost-like environment, such as food utensils, PHBV films used for food preservation, and blow-molded straws typically made of P(3HB-co-3HHx). Compared to polyethylene-lined paper cups, PHA-based coatings may offer recyclable alternatives [3]. PHAs are also applicable for lightweight cushioning foams, latex-like products, and heat-sensitive adhesives for use in cosmetics and labeling (Table 2) [95].

#### PHA in textile innovation

PHA can be turned into fibers similarly to nylon, mitigating the drawbacks of static electricity and low air permeability of regular polyester materials (Fig. 3). Suitable for various textile applications, PHA can potentially replace >50% of fossil polymers now used in textiles [96]. By employing advanced drawing and annealing techniques, proper additives to various copolymeric compositions, and blending with other biopolymers help enhance its mechanical qualities, thus the drawbacks of PHA (high production cost, inherent brittleness, and low processing window) could be resolved [106]. For example, polylactic acid (PLA)/PHA blends can reduce the aging and secondary crystallization of both polymers. Meanwhile, medical surgical garments, upholstery, and carpets have been fabricated to achieve the full potentials of PHA in the textile industry (Table 2) [95].

#### PHA for cosmetics and antimicrobial products

PHAs have been developed for use in the cosmetics and personal care industries, being utilized in various products including beauty masks, cosmetic toner and facial pads, as well as cast films (Fig. 3) [95]. Microplastics from cosmetics, toothpaste, and facial cleansers enter aquatic ecosystems and may accumulate in seafood, ultimately posing health risks to humans through dietary exposure [107]. PHA microbeads are commercially exploited in sunscreen to offer UV protection, and in skin peeling and scrubbing, replacing conventional primary light scattering microplastic (typically of PE) [96]. One of the first examples of PHA used in cosmetics are sunscreen products containing fully biodegradable PHA micropowders (brand name: MyKAI) developed by Unilever. Multiple PHA microplastic-free technologies are under development [108]. Furthermore, it has been reported that the excellent antibacterial properties of low-molecular-weight PHAs have increasingly aroused interest in their use for sanitary products, including diapers, feminine hygiene products, and cosmetic containers (Table 2) [109].

#### Medical applications of PHA

The excellent biodegradability and biocompatibility of PHAs facilitate their use in tissue engineering and several biomedical-related sectors (Fig. 3). PHA can be developed into tissue-engineered scaffolds or microspheres to restore, maintain, or improve soft and hard tissues, including bone, cartilage, skin, nerves, muscle, and blood vessels systems [100]. PHAs have been extensively studied for the manufacturing of efficient drug delivery systems like microdevices, nanodevices, discs, implants and rods and films, to achieve the localized and targeted controllable release of anticancer drugs, immunotherapy drugs, growth factors, and to deliver antigen carriers and bionic vaccines [100]. Additionally, approval of PHA clinical products has also been gained in the USA and Europe, such as absorbable sutures for tissue repair and reconstruction. Notably, P4HB is the first and only PHA approved by the FDA for medical applications so far (Table 2) [100].

#### Environmental remediation and agriculture

The denitrification of water and wastewater is one of the prospective uses of PHA, offering benefits over conventional nitrogen removal methods with excellent performance: PHA as a microbial substrate enables more efficient targeting of nitrogen and organic waste removal from water systems, reducing excess carbon addition and improving effluent quality (Fig. 3) [98]. PHA mulching films have been used to prevent soil pollution and retain water to facilitate plant growth; depending on climate conditions, PHA can be degraded in soil over time [95]. PHA can also be used as coating materials for urea fertilizers, herbicides, and insecticides to achieve controlled release and improve efficiency (Table 2) [98].

#### PHA uses in aquaculture applications

PHA application has been extended to the aquaculture industry (Fig. 3). Particularly, the short-chain type PHB can be used as an effective feed additive and biocontrol agent in aquaculture to improve growth and in disease prevention [110]. Recently, it has been demonstrated that PHB can increase the growth and survival of large yellow croaker fish, *Macrobrachium rosenbergii* prawn, juvenile European sea bass, Chinese mitten crab (*Eriocheir sinensis*), zoea larvae, and blue mussel (*Mytilus edulis*) larvae [111–115]. On the other hand, PHB can be applied as an effective biocontrol agent and an immunostimulant for aquatic species, replacing soluble smelling short-chain fatty acids and antibiotics. In addition, PHB could be later biodegraded into its intermediates in the animal gut, such as butyrate and 3-HB monomers and oligomers, to inhibit pathogens and improve the growth and immune system of aquatic animals (Table 2) [116].

## PHA BIODEGRADATION

**Figure 4.**
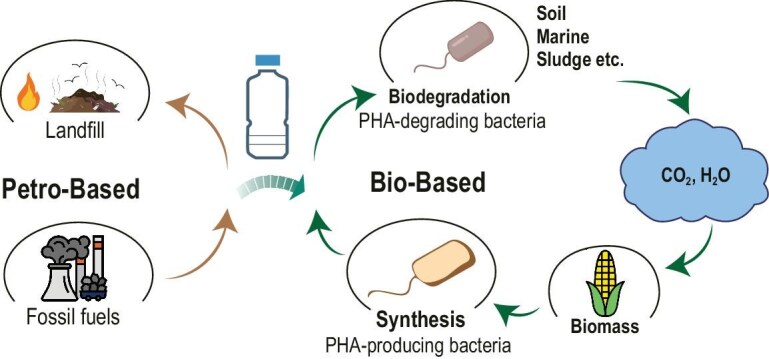
Life cycle of biodegradable and non-biodegradable plastics [10,117–122].

For the large-scale deployment of PHA, comprehensive life cycle management must be addressed, with a particular focus on efficient degradation pathways in both natural ecosystems and engineered environments, as well as effective post-consumer waste treatment technologies [117]. The complete decomposition of PHA is crucial for its reintegration into the natural biogeochemical cycle (Fig. 4) [117].

Bioplastics that are capable of undergoing biodegradation are distinct from biobased plastics derived from biomass such as plants, trees, and algae. However, many biobased plastics—including PE, PP, polyamides (PAs), and PET are non-biodegradable and pose environmental threats similar to conventional plastics [118,119]. Biobased compostable plastics such as PLA require specific thermal and pressure conditions for degradation, typically achievable only in industrial composting facilities [120,121]. In contrast, truly biodegradable materials—such as certain PHAs—can be broken down by microorganisms into benign end-products such as water and carbon dioxide together with microbial biomass, under both natural and engineered environmental conditions [122].

### PHA biodegradation mechanisms

**Figure 5.**
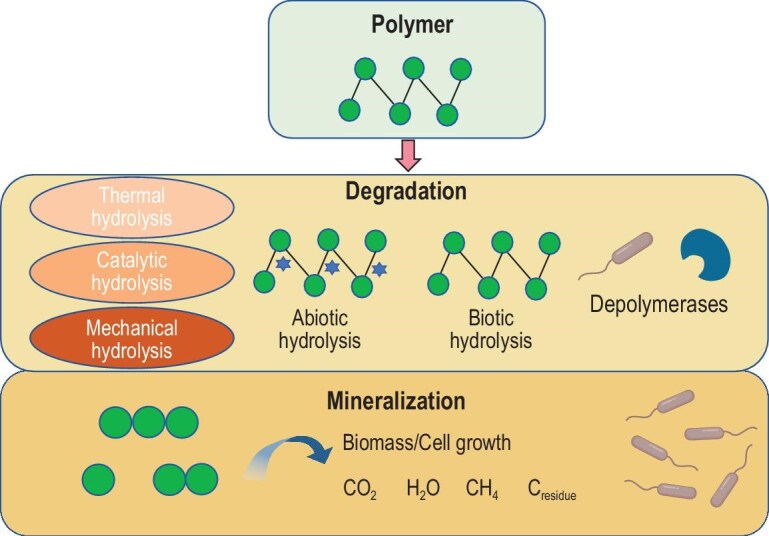
Degradation process of biodegradable polymer materials [117,119,123–130]. It involves two steps: (1) the degradation of the polymer main chain, and (2) the mineralization of decomposed products during catabolism.

The degradation mechanisms of biobased polymers can be divided into two stages: abiotic degradation and biodegradation (Fig. 5) [123]. Among biobased polymers, only PLA and PHA are fully biobased and can undergo biodegradation in some form, a process that is highly dependent on the action of microorganisms and enzymes [119].

Physical and chemical processes such as photooxidative degradation, thermal degradation, and mechanical degradation can alter the structure and surface properties of PHA, thereby increasing the surface area available for microbial contact and initiating or enhancing biodegradation [1]. In the case of photooxidative degradation under ultraviolet (UV) irradiation, the absorption of light energy promotes the formation of free radicals within the polymer chain, which can induce chain scission. However, this process is dependent on the intensity and duration of UV exposure [117]. Thermal degradation relies on high-temperature conditions to cause random chain scission in PHA polymer chains, splitting them into smaller units such as oligomers, acids, alcohols, esters, and radicals [1]. While thermal degradation can enhance other forms of degradation through increased collisions between reactants and enzymes, its practical application is limited by the generation of by-products and high energy requirements [124]. Mechanical degradation involves the application of physical forces to break down PHA polymers. This method primarily results in physical fragmentation of the polymer rather than chemical decomposition at the molecular level [125]. Consequently, mechanical degradation only achieves polymer fragmentation without complete molecular deconstruction [125]. Catalytic degradation accelerates the breakdown of PHA by utilizing catalysts such as enzymes, transition metal ions, or nanozymes. These catalysts specifically target the ester bonds in PHA, yet the reliance on specific catalysts can increase costs and complicate the process [126].

Microorganisms degrade macromolecules mainly through extracellular processes, such as the secretion of extracellular depolymerase that degrade PHA polymers into CO_2_, H_2_O, CH_4_, and form new biomass, which can be used by various microbial communities as metabolic intermediates for their growth and as a source of energy [127]. Biodegradation occurs through four primary stages: (1) bio-deterioration, during which the chemical and physical properties of the polymer are altered; (2) bio-fragmentation, involving the breakdown of the polymers into simpler forms; (3) assimilation, where microorganisms take up polymer molecules and turn them into their cell biomass; and (4) mineralization, characterized by the production of oxidized metabolites following the degradation process (Fig. 5) [128–130].

These microorganisms are widely distributed in soils, aquatic environments, and extreme conditions. The efficiency of enzymatic degradation is influenced by factors such as polymer composition, crystallinity, environmental conditions, and possibly, in the presence of cofactors [117].

### Diversity of PHA-degrading microbes in various environments

Numerous studies have documented the molecular organization and enzymatic characteristics of various PHA-degrading microorganisms and their associated depolymerases, as well as the degradation behavior of PHA in diverse environments such as freshwater, soil, wastewater, and activated sludge [131,132]. The degradation kinetics of PHA products are influenced by several factors, including the chemical composition of PHA, the size and shape of PHA products, the structure of microbial communities, climatic conditions, and the presence of additives introduced during PHA processing [133,134]. PHA is hydrolyzed by microbial-secreted depolymerases into oligomers or monomers such as R-3-hydroxybutyrate and R-4-hydroxybutyrate [88]. These monomers are subsequently metabolized via the β-oxidation pathway or the tricarboxylic acid (TCA) cycle into CO_2_ and H_2_O, forming microbial biomass under aerobic conditions, or methane (CH_4_) under anaerobic conditions [135,136]. The degradation products are non-toxic and participate in the natural carbon cycle, potentially improving soil fertility or water quality [135,137]. While degradation efficacy is well-demonstrated in controlled laboratory settings, degradation rates in real-world environments (e.g. landfills or soil) are significantly lower due to uncontrollable environmental variability [137,138].

### PHA biodegradation in soil

#### Influence of soil sources

Soil environments harbor diverse PHA-degrading microorganisms represented by various species of *Pseudomonas, Bacillus*, and/or *Streptomyces* serving as core functional genera driving the degradation process (Fig. 4) [117]. The efficiency of PHA biodegradation is significantly enhanced when exposed to aerobic conditions in surface soil layers, though this process is modulated by regional climate, temperature, and moisture. For example, sandy soils exhibit slow degradation due to poor water availability, whereas agricultural soils with high microbial diversity (e.g. *Burkholderia, Cupriavidus*) enable complete PHA degradation within 28 days [1]. Soil pH indirectly regulates degradation by influencing enzymatic activity, with an optimal range of 6–9 [135]. For instance, saline-alkali soils (pH 7–8) facilitate faster PHB degradation compared to acidic clay soils. However, ∼50% of global top soils have pH <6.5, reducing the efficiency of natural degradation [139].

Notably, high-efficiency degraders persist in extreme environments such as cold regions [3]. In Siberian permafrost, bacterial genera (*Bacillus, Pseudomonas*) and fungal taxa (*Aspergillus, Penicillium*) demonstrate robust degradation capabilities toward PHB films, as evidenced by molecular weight analysis and scanning electron microscopy (SEM) [140]. These microbial communities achieve sustained low-temperature degradation through adaptive strategies, including the secretion of cold-active enzymes, reduced metabolic demands, or structural optimization of depolymerases [117]. These findings highlight the habitat-specific composition of microbial consortia and their degradation activity, which is closely linked to local environmental adaptation.

#### Microbial functionality and polymer structure

The PHA biodegradation rate is highly dependent on its chemical structure. For instance, the homopolymer PHB typically degrades more slowly than copolymers such as PHBV with lower crystallinity [133]. This difference is largely attributed to the substrate specificity of microbial depolymerases. Some strains, such as *Priestia aryabhattai*, are capable of degrading various PHA types, although the degradation efficiency varies significantly depending on the polymer composition [117]. In particular, species of the genus *Pseudomonas* demonstrate strong PHA-degrading abilities in composting environments, primarily through the secretion of specialized extracellular depolymerases. These organisms play a pivotal role in the microbial degradation of bioplastics, offering great promise for waste management and sustainable recycling systems [141].

### Biodegradation of PHA in marine environments

In addition to terrestrial soil environments, the marine environment also serves as a unique habitat for PHA-degrading microorganisms (Fig. 5). Notably, compared to PLA, PHA is the only biobased polymer that meets ASTM standards and can effectively be degraded in marine environments, including coastal, shallow-sea, and deep-sea environments [142]. The degradation products of PHA are metabolized and mineralized by marine microorganisms, thereby completing the carbon cycle [119]. Thus, PHA not only participates in the carbon cycle in terrestrial environments but also plays a significant role in marine ecosystems [142]. The biodegradation of PHA has been confirmed across diverse marine environments, and PHA-degrading bacteria have been isolated from various marine environments where PHA degradation occurs [119,142].

Compared to PHA-degrading microorganisms in terrestrial environments, their marine counterparts exhibit remarkable adaptability to high salinity and specialized ecological niches, demonstrating sophisticated physiological traits and functional diversity [117]. These microbes not only thrive in PHA degradation under specific salinity and temperature regimes but also display enhanced depolymerase activity under optimal conditions, positioning them as vital resources for combating marine plastic pollution [133,142]. Critical genera such as *Enterobacter, Bacillus, Comamonas*, and *Gracilibacillus* have been identified as dominant contributors to PHA breakdown in seawater and marine sediments [135]. Notably, unique PHA-degrading strains—including *Acidovorax* spp. isolated from Siberian sturgeon and *Acinetobacter* spp. sourced from European sea bass—highlight the ecological versatility of marine microbial communities participating in marine plastic biodegradation.

The degradation rate of PHA in marine environments is governed by a combination of factors, including microbial density, temperature, and material crystallinity [90,96,100]. Studies indicate that hydrodynamic forces and ecological diversity in marine systems enhance microbial-PHA interactions, facilitating degradation while microbial populations in seawater are generally lower than those in soil or freshwater [133,142]. A critical accelerator of this process is the formation of a specialized microbial biofilm termed the ‘plastisphere’ on the PHA surface [142]. This microenvironment enriches PHA-degrading microbial communities (e.g. *Pseudomonas* and *Marinobacter* spp.), thereby accelerating enzymatic hydrolysis through localized enzyme secretion and substrate accessibility [142]. Furthermore, a positive correlation exists between PHA biodegradation rates and ambient temperatures within the range of 10–27°C [119].

PHAs also exhibit varying degradation rates in marine environments, influenced by their structural properties and environmental conditions [143]. PHB homopolymer, characterized by high crystallinity, degrades relatively slowly due to limited enzymatic accessibility [144]. Field tests in coastal Japan showed a 60% weight loss after 2 weeks. In contrast, PHBV copolymers of P(3-hydroxybutyrate-co-3-hydroxyvalerate) display reduced crystallinity (∼29%) from valerate incorporation, significantly accelerating degradation [145]. PHBHHx, with even lower crystallinity (∼20%) from 3-hydroxyhexanoate units, demonstrates the fastest degradation [119,145]. The presence of larger amorphous regions enhances enzymatic hydrolysis, resulting in accelerated mechanical disintegration despite minimal reductions in molecular weight [119].

### Biodegradation of PHA in sewage sludge

#### Degradation mechanisms

While sewage sludge is not a typical PHA source, mesophilic anaerobic degradation (<40°C) enables simultaneous waste treatment and energy recovery [54,98]. Following ASTM D5511, PHA mineralizes completely within 20 days, producing CO_2_ and methane [135]. Its linear structure doubles enzymatic hydrolysis rates compared to PLA/polycaprolactone (PCL) [146]. The degradation product 3-hydroxybutyrate (3HB) configuration directs metabolic flux toward methane synthesis, with PHB generating biogas containing 80% methane at 60% carbon conversion efficiency. In addition, environmental condition modulation critically affects degradation efficiency. Under simulated high-solids landfill conditions (>50% solids content), PHB demonstrates accelerated degradation with complete mineralization within 9 days [135]. However, PHBV shows significantly reduced degradation rates due to crystalline structure complexity induced by copolymer units.

#### Temperature-dependent degradation dynamics

PHA degradation exhibits strong thermal sensitivity within sewage sludge systems. Mesophilic conditions (30–40°C) optimize enzymatic activity, as evidenced by ISO 13 975 data: PHB achieved 90% degradation in 10 days at 37°C vs 22 days for 83%–98% degradation at 55°C. This contrasts with PLA/PCL, which require thermophilic temperatures (>37°C) for significant breakdown, highlighting PHA adaptability to natural anaerobic environments [147].

#### Inoculum specificity and co-digestion synergy

The microbial consortia within sewage sludge critically determine PHA degradation efficiency [117]. *Pseudomonas* spp. and *P. aryabhattai* isolated from activated sludge exhibit 1.8-fold higher depolymerase activity compared to freshwater sediment communities [117]. This functional superiority stems from their evolutionary adaptation to sludge heterogeneous organic matrices, where lipase-box motifs in depolymerases (Gly-Ile-Ser-Ala-Gly) stabilize enzyme-substrate interactions under fluctuating redox conditions [117]. While methane yield increases due to complementary carbon sources [148], labile substrates like glucose trigger catabolite repression [149]. Furthermore, *P. aryabhattai* exhibits metabolic flexibility, shifting from PHA hydrolysis to glucose utilization without complete repression, suggesting strain-specific regulatory pathways exploitable for process optimization (Fig. 5) [117].

### Perspectives of PHA biodegradation

PHAs not only reduce greenhouse gas emissions but also address mismanaged plastic waste in environments where human intervention is ineffective (e.g. soil, marine systems) [10]. Their biodegradation is driven by bacterial and fungal enzymes, producing non-toxic metabolites that integrate into natural carbon cycles, enhancing soil fertility and marine ecosystems. PHAs outperform other biopolymers (e.g. PLA, poly(butylene adipate-co-terephthalate) or PBAT) in aerobic environments (soil, compost, marine) and exhibit promising degradation in anaerobic systems (landfills, sewage sludge) [136].

However, challenges persist. To transition PHA from laboratory potential to scalable industrial applications, depolymerases could be engineered for enhanced performance under diverse environmental and operational conditions. Critical enzyme properties include fast catalytic kinetics, long-term stability, and adaptability to stressors such as high temperatures, salinity, and detergents. Extremophile microorganisms thriving in extreme environments represent an untapped reservoir for discovering robust depolymerases with unique thermal or chemical resilience [135]. However, progress is hindered by limited structural insights; the absence of high-resolution 3D enzyme architectures obstructs mechanistic studies of substrate binding, catalytic efficiency, and stability under non-ideal conditions [117]. Blending PHA with additives (e.g. Joncryl, hydrophobic plasticizers) or non-degradable polymers often inhibits biodegradation, particularly in single-use consumer plastics [135]. Conversely, incorporating natural fibers (e.g. starch, proteinaceous materials, or cellulose) enhances hydrophilicity and accelerates biodegradation [135]. Tailoring PHA crystallinity and surface area through processing techniques or blending with hydrophilic natural fibers to enhance enzyme interaction and hydrolysis rates may be an effective approach.

In addition, establishing globally recognized protocols for evaluating biodegradation across diverse environments to ensure reliable data for policymakers and industries is also important. By addressing these gaps, PHA can transition from niche applications to mainstream alternatives, aligning with global efforts to establish a circular bioeconomy.

## LIFE CYCLE OF PHA

### Toward sustainable PHA production by engineered *Halomonas*

Recent advances in genetic engineering of *H. bluephagenesis* have demonstrated significant cost reductions in PHA production through targeted chromosome modifications (Table 3). Park *et al.* achieved a 40% reduction in γ-butyrolactone usage and a 50% decrease in shear stress by knocking out exopolysaccharides (EPS), which also enabled 10-fold faster centrifugation (15–30 min), 30% less water consumption, and 35% fewer enzymes for purification [69]. Chen *et al.* further streamlined downstream processing (DSP) by developing an autolytic system that eliminated lysozyme use (100% reduction) and reduced water consumption by 30.7%, while improving PHA purity to 99.2% [29]. In another study, Chen *et al.* engineered cell morphology to increase PHB storage capacity, reducing centrifugation energy requirements by 50% (from 8000 rpm to 4000 rpm) and lysozyme usage by 28% [22]. Zhang *et al.* addressed upstream costs by engineering low-salt-tolerant strains, achieving a 40% reduction in carbon source consumption, 30% less water usage, and 70% lower wastewater treatment costs due to reduced effluent salinity [150]. Collectively, these modifications yielded an estimated 33% reduction in total production costs (Table 3).

**Table 3.** Genetic strategies and their economic benefits.

Manipulation	Catalogues	Cost Reduction	Description	Reference
EPS knockout	Expensive substrate	∼40%	Reduced high-cost γ-butyrolactone (40%)	[69]
	Energy	∼50%	Reduced rotation speed (50%)	
	Segragation time	∼90%	centrifugation time decreased tenfold due to rapid cell self-flocculation (15–30 min)	
	Water	∼30%	-	
	Enzyme usage	∼35%	-	
Autolytic system (T4 holin/endolysin)	Enzyme usage	100%	100% reduction in lysozyme usage	[29]
	Downstream extraction time	∼26%	-	
	Extraction, Water	∼30.7%	-	
Morphology engineering (MreB degradation)	Energy	∼50%	Lower centrifugation speed (4000 rpm vs. 8000 rpm)	[22]
	Enzyme usage	∼28%	Reduced lysozyme (28%)	
Low-salt tolerance (ARTP mutagenesis)	Material	∼40%	Reduced carbon (40%), steam (30%), wastewater treatment (70%)	[150]
	Water	∼30%	Less extraction and purification water usage	
	Energy	∼40%	Less washing cycle	
	Steam	∼30%	Reduced evaporation	
	Wastewater	∼70%	Lower NaCl concentration	
*etf* operon knockout (self-flocculation)	Energy, Wastewater	-	No centrifugation, reusable supernatant, “wastewaterless” process	[151]
Morphology engineering (*mreB*/*ftsZ*)	Energy, Extraction	-	Spherical/elongated cells enabled low-energy sedimentation	[81]

For *H. campaniensis* LS21, cost-saving strategies focused on self-flocculation and morphology engineering. Ling *et al.* engineered self-flocculating strains by deleting the electron transfer flavoprotein (*etf)* operon, enabling biomass collection via siphoning and eliminating centrifugation costs [151]. This approach also facilitated a ‘wastewaterless’ process, reusing supernatant for consecutive batches, and improved productivity to 0.33 g/L/hr PHB. Jiang *et al.* engineered spherical and elongated cell morphologies, increasing PHB titers by ∼80% and enabling low-energy separation due to rapid sedimentation [81]. These modifications achieved glucose-to-PHB conversion rates of 42%–44%, nearing the theoretical maximum (Table 3).

In summary, genetic engineering of *Halomonas* spp. has reduced PHA production costs by >33% through optimized DSP, reduced resource consumption, and improved process efficiency [22,29,69,141]. These advancements highlight the potential of tailored strain modifications for sustainable and cost-effective biomanufacturing (Table 3).

### Life cycle assessment of PHA

The global warming potential (GWP) of PHAs serve as a key metric for evaluating their environmental performance. GWP values vary considerably depending on the feedstock source and energy configuration of the production process [152]. A seminal study calculated that sucrose-based PHB has a GWP of 1960 kg CO_2_-equivalent per 1000 kg polymer, in contrast to 3530 kg CO_2_-equivalent for fossil-based polypropylene (PP)—a reduction of nearly 50%, which exhibits a notably lower GWP compared to conventional plastics [152]. The use of waste-derived feedstocks further improves this profile: employing waste glycerol from soybean oil processing can result in a net negative emission of −2.52 kg CO_2_ per kg of PHA, as this route avoids emissions generated by conventional waste disposal [153].

Regarding Non-Renewable Energy Use (NREU), the PHA production process—particularly fermentation and extraction—is energy-intensive. Nevertheless, the biomass origin of the feedstock reduces dependence on fossil resources. Sucrose-based PHB shows an NREU ranging between 40 and 50 MJ/kg, significantly lower than the 85.9 MJ/kg required for PP, due in part to the use of bagasse as an on-site energy source [152,154]. This advantage is even more pronounced when using soybean oil, with an NREU as low as 4.8 MJ/kg [153].

First-generation PHA feedstocks involve trade-offs in land, water, and agricultural impacts. Land use is estimated at 0.23 hectares per ton of PHA [155]. Water consumption averages 40 m^3^ per tonne of PHA during extraction and processing, though this value is susceptible to regional water availability and irrigation demands [155]. In terms of emissions contributing to eutrophication and acidification, producing 1000 kg of PHA results in ∼5.19 kg PO_4_-equivalent and 24.9 kg SO_2_-equivalent, which compares favorably to PP at 5.84 kg PO_4_-equivalent and 448.8 kg SO_2_-equivalent [152].

End-of-life behavior also influences the sustainability profile of PHA. In marine environments at 25°C, 28-day biodegradation tests based on biochemical oxygen demand reported degradation rates of 14%–27% for P(3HB), 78%–84% for PHBV, and 43%–51% for P(3HB-co-4HB) [156]. However, if mixed with conventional plastics such as PET, PHA can compromise the quality of recycled materials. Thus, effective separation, collection, and labeling systems are necessary to prevent contamination of recycling streams [157].

In summary, life cycle assessment reveals PHA as a polymer class with multifaceted environmental implications. While it shows clear potential in reducing fossil carbon dependence and greenhouse gas emissions, its overall benefit is closely tied to feedstock type, process energy sources, and waste management pathways [158,159]. Moving forward, priorities should include advancing waste-based feedstocks, improving energy efficiency in production, and developing a dedicated organic recycling infrastructure [157,160]. Such measures are essential to maximize the environmental benefits of PHA while minimizing pressure on agricultural land and water resources.

## CONCLUSIONS AND PERSPECTIVES

Synthetic biology has profoundly transformed both the research and industrial production of PHA. By reprogramming microbial chassis, optimizing metabolic pathways, and adopting NGIB based on halophilic bacteria, significant progress has been achieved in the customized synthesis and scalable production of PHA [17,161,162]. The halophile-based NGIB utilizes seawater as a medium, enabling continuous non-sterile open fermentation for >65 days with a high cell density [17,83], while the self-flocculating separation technology reduces downstream processing costs [69]. The combination of synthetic biology tools and morphological engineering is expected to further improve cell density to 200 g/L, increasing PHA content beyond 80 wt%, and improving conversion rates to 50%, thereby driving cost reduction [8,163]. However, current production costs and material performance of PHA remain uncompetitive compared to conventional plastics, necessitating breakthroughs in addressing poorly characterized genomes of chassis strains, insufficient metabolic insights, and the development of cost-effective downstream processes to establish a closed-loop green production system. Finally, scale also decides the production cost of PHA.

Regarding PHA production, substrate selection and process optimization are pivotal for life cycle design. *Halomonas* can utilize non-food substrates such as lignocellulosic hydrolysates, food waste, and acetate [35,39], reducing reliance on grain-based resources. Coupled with seawater-based fermentation and renewable energy (e.g. wind or solar power), this approach promotes sustainable offshore or island seawater-based biomanufacturing [34]. Advances in high-density cultivation—such as directed evolution of oxygen-tolerant enzymes and rational design of salt-resistant components—will enhance production efficiency. Additionally, downstream innovations such as the *etf* operon knockout in the self-flocculating strain *Halomonas* LS21 and the development of low-salt-tolerant mutants via mutagenesis help mitigate issues related to high-salinity wastewater discharge and equipment corrosion [34]. These advances are critical to enabling green and closed-loop bioprocesses.

Diversification and functionalization of PHA properties are central to expanding their applications. Chemical modification of side chains, microstructure regulation, and AI-driven production lines can yield PHA with enhanced thermal stability, mechanical strength, and biocompatibility [85]. Nevertheless, intrinsic limitations in thermodynamic performance hinder high-value applications, calling for interdisciplinary strategies (e.g. materials science and computational biology) to develop novel functional monomers or composite systems. Blending PHA with hydrophilic natural fibers (e.g. starch, cellulose) accelerates degradation rates [135], while avoiding non-degradable additives is crucial for single-use plastic alternatives.

In waste management and environmental impact mitigation, the biodegradability of PHAs position them uniquely within circular economies. Microbial enzyme-driven degradation integrates PHA metabolites into natural carbon cycles, reducing the risks of plastic pollution in soil and marine ecosystems [136]. However, optimizing depolymerase efficiency and environmental adaptability remains challenging. Mining extremophiles for robust enzymes and resolving their 3D structures are key priorities. LCAs confirm PHA superiority in fossil fuel displacement and carbon reduction [164], yet further optimization of substrate sourcing, wastewater recycling, and energy consumption is essential to achieve the ‘design-regenerate-redesign’ paradigm.

Future industrialization of PHA hinges on three fronts: (1) advancing synthetic biology tools for halophiles and extremophiles to develop universal chassis and efficient metabolic networks for low-cost PHA synthesis; (2) fostering interdisciplinary innovation (e.g. AI, materials chemistry) to enhance material performance and smart manufacturing; and (3) establishing life cycle management frameworks, including standardized degradation protocols, greening of waste, and circular economy models. With technological advancements and policy support, PHAs hold immense potential to transition from laboratory-scale production to mainstream biomaterials, addressing global plastic pollution and advancing sustainable development goals.
